# TCTN2: a novel tumor marker with oncogenic properties

**DOI:** 10.18632/oncotarget.20438

**Published:** 2017-08-24

**Authors:** David Cano-Rodriguez, Susanna Campagnoli, Alberto Grandi, Matteo Parri, Elisa De Camilli, Chaojun Song, Boquan Jin, Aurelien Lacombe, Andrea Pierleoni, Mauro Bombaci, Chiara Cordiglieri, Marcel HJ Ruiters, Giuseppe Viale, Luigi Terracciano, Paolo Sarmientos, Sergio Abrignani, Guido Grandi, Piero Pileri, Marianne G. Rots, Renata Grifantini

**Affiliations:** ^1^ Externautics SpA, Siena, Italy; ^2^ Department of Pathology and Medical Biology, University of Groningen, University Medical Center, Groningen, The Netherlands; ^3^ Department of Pathology, European Institute of Oncology, Milan, Italy; ^4^ Department of Immunology, The Fourth Military Medical University, Xi’an, China; ^5^ Institute of Pathology, University Hospital Basel, Basel, Switzerland; ^6^ Department of Oncology and Hemato-Oncology, University of Milan, Milan, Italy; ^7^ Istituto Nazionale Genetica Molecolare, Padiglione Romeo ed Enrica Invernizzi, IRCCS Ospedale Maggiore Policlinico, Milan, Italy; ^8^ Centre for Integrative Biology - CIBIO, University of Trento, Trento, Italy; ^9^ Present affiliation: European Bioinformatics Institute (EMBL-EBI), Wellcome Genome Campus, Hinxton, Cambridge, UK

**Keywords:** TCTN2, cancer, biomarker, oncogene, epigenetic editing

## Abstract

Tectonic family member 2 (*TCTN2*) encodes a transmembrane protein that belongs to the tectonic family, which is involved in ciliary functions. Previous studies have demonstrated the role of tectonics in regulating a variety of signaling pathways at the transition zone of cilia. However, the role of tectonics in cancer is still unclear. Here we identify that TCTN2 is overexpressed in colorectal, lung and ovary cancers. We show that different cancer cell lines express the protein that localizes at the plasma membrane, facing the intracellular milieu. TCTN2 over-expression in cancer cells resulted in an increased ability to form colonies in an anchorage independent way. On the other hand, downregulation of TCTN2 using targeted epigenetic editing in cancer cells significantly reduced colony formation, cell invasiveness, increased apoptosis and impaired assembly of primary cilia. Taken together, our results indicate that TCTN2 acts as an oncogene, making it an interesting cancer-associated protein and a potential candidate for therapeutic applications.

## INTRODUCTION

Cilia are microtubule-based organelles extending from the cell surface, which play a pivotal role in eukaryotic biology. Primary (non-motile) cilia have an important mechanical and chemical sensory role since they regulate several signal transduction pathways, including the Wnt, Hedgehog (Hh), Platelet-Derived Growth Factor Receptor alpha (PDGFRα) and mTOR signaling systems [1–7]. Primary cilia sense a wide variety of extracellular signals to control decisions regarding development, proliferation, differentiation and tissue maintenance. Impairment of ciliary function results in a number of diseases, known as ciliopathies, such as polycystic kidney disease (PKD), Nephronophthisis (NPHP), Alström syndrome (ALS), Bardet-Biedl syndrome (BBS), Joubert syndrome (JBTS), Meckel-Grüber syndrome (MKS) and others [8–10]. In line with the role of cilia in several cellular functions and signaling pathways, recent studies have also highlighted the importance of cilia in the development of cancer. Alteration or loss of primary cilia has been correlated with cellular transformation. Accumulating evidences indicate that cancer cells lacking cilia show a reduction or modification in their response to extracellular signals that regulate growth and differentiation [11–13]. Indeed, a number of critical cell signaling and adhesion molecules are clustered in the cilium, and require an intact cilium for normal function [3, 5, 14]. These include components of the Shh and Wnt signaling pathways, whose activation/inactivation has been linked to several types of cancer [15]. Effectively, ciliated tumors may have either active Hh or Wnt [12, 16, 17]. On the other hand, disruption of sensory cilia leads to inhibition of tumorigenesis, due to inactivation of Hh signaling [13, 18].

Within ciliary protein components, there are members of the tectonic family (TCTN1, TCTN2, TCTN3) that have been identified as important regulators of Hh pathway [19, 20]. Mutations of the tectonic proteins are associated to ciliary dysfunction that underlies several ciliopathies [10, 21]. Tectonics (TCTNs) form complexes with proteins that localizes to the ciliary transition zone, where they regulate ciliogenesis and ciliary membrane composition in a tissue-dependent manner [22]. Concerning the role of TCTNs in cancers, available information are limited to TCTN1, which has been described as an important regulator of proliferation and migration of cancer cells [23]. TCTN1 is also important to maintain active Hh signaling during neural tube development [19]. Tectonics could exert their activity in signal transduction through their functional interaction with MKS1, a ciliary basal body protein that has a potential role in regulating both Wnt signaling, which regulates cell proliferation and its alteration causes different diseases, including cancer. MKS1 also acts upstream of Patched and influences Shh signaling. Loss of MKS1 causes a reduction in high-level Shh signaling. In various types of tumor, mutation in Hedgehog signaling leads to hypoplasia [24], thereby inhibition of the Shh, one of the major components of the pathway, has been identified as a possible therapeutic strategy for gastric cancer [25].

In this study, we report for the first time the presence of TCTN2 in a significant percentage of colorectal cancer (CRC) as well as lung and ovary cancers. Moreover we find the protein to be expressed at the level of the plasma membrane in cancer cell lines of different origins. By using a gene-targeting approach, we also show that inhibition of TCTN2 expression significantly affects cell invasiveness, clonal growth, cilia formation, and increases cell apoptosis. Conversely, TCTN2 over-expression increases the clonogenic property of cancer cells. All these data together suggest that TCTN2 could be a novel oncogene and that it could be exploited as potential candidate for therapeutic applications.

## RESULTS

### TCTN2 is over-expressed in human cancers

**Figure 1:**
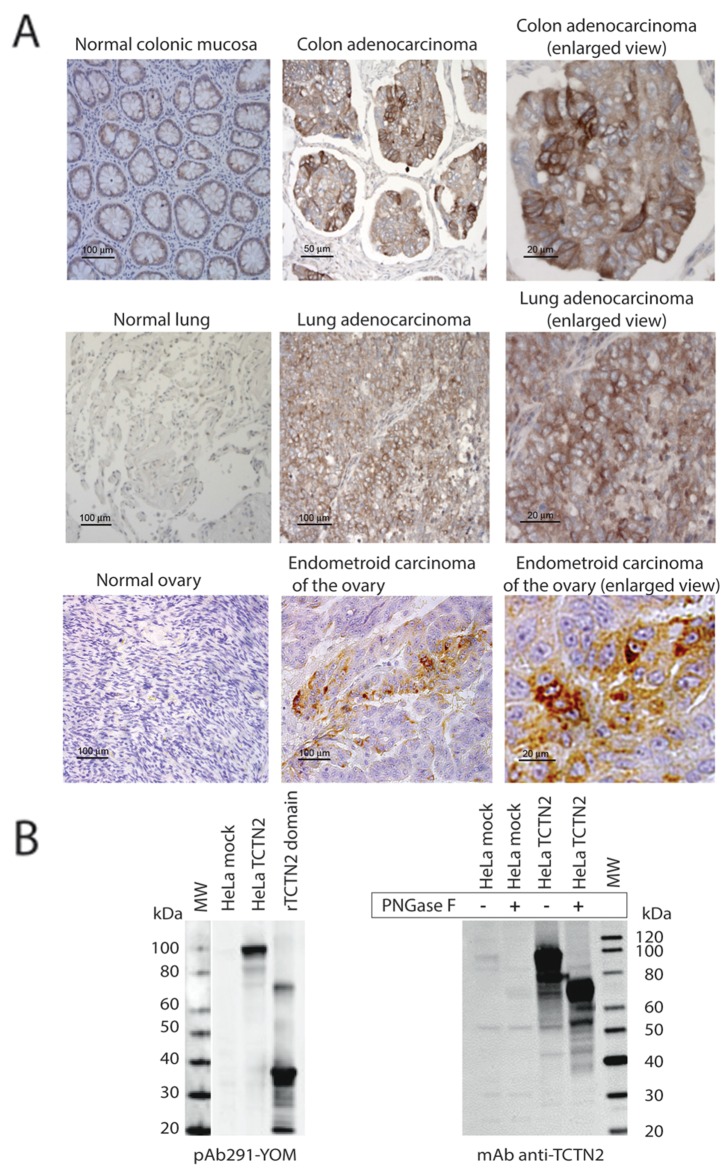
TCTN2 is overexpressed in primary tumors of colon lung and ovary **(A)** TCTN2 is overexpressed in tumor samples and localizes at the cell membrane. Cancerous and matched normal samples from colon, lung and ovary were stained with the anti-TCTN2 monoclonal antibody, arrayed in parallel on the same TMA slides and analyzed simultaneously. **(B)** Specific recognition of TCTN2 antibodies - TCTN2 expressed in mammalian cells is a glycosylated protein. Western blot on total protein extracts from HeLa cells transfected with the empty plasmid or full length TCTN2, or recombinant TCTN2 domain (amino acids 171-444) expressed in E. coli, using the polyclonal (pAb291-YOM, left panel) and monoclonal antibody (anti-TCTN2 mAb, right panel). The recognition of the mAb anti TCTN2 was also assessed after treatment with Peptide-N-Glycosidase F (PNGaseF).

In our recent research, we described the use of the YOMICS^®^ murine polyclonal antibody library (http://www.yomics.com) to discover tumor markers by IHC analysis [26, 27]. In essence, this antibody library includes antibodies raised against 1287 secreted or membrane-associated human proteins, most of which are still marginally characterized. The entire antibody library is currently used to screen Tissue microarrays (TMAs) carrying clinical samples from major human cancers. During the screening of this antibody library on TMAs containing cancerous and normal formalin-fixed paraffin-embedded (FFPE) samples from breast, colorectum, lung, ovary and prostate (5 tumor and 5 matched normal samples, in duplicate), we found that a polyclonal antibody (pAb291-YOM) raised against a recombinant domain of TCTN2, encompassing the protein region from amino acid 170 to amino acid 443 (rTCTN2), specifically detected the expression of its target protein in cancer samples of colon (3/5), lung (2/5) and ovary cancers (2/5) whereas it gave a negligible staining in the corresponding normal tissues (Supplementary Figure 1). Based on this initial indication, a mouse monoclonal antibody (TCTN2 mAb) was generated in-house against the same domain (rTCTN2) utilized to generate the polyclonal sera and used to analyze approximately 50 samples from colorectum, lung and ovary cancers, and 5 samples from matched normal tissues, in duplicate. In general the monoclonal antibody staining was cytoplasmic, though in some samples it also decorated the plasma membrane (Figure 1A). This mAb mainly detected TCTN2 in colorectum cancer (63.8%; P value <0.0001), as compared to lung (18%), and ovary (16%) cancer samples, with marginal or negative staining in tested normal samples. An IHC scoring system based on a combined evaluation of the intensity of mAb staining (from 1 to 3) multiplied by the percentage of positive cancer cells (from 1 to 100), showed that a higher fraction of samples with strong or moderate staining (IHC score >100) in colorectal (30%) vs lung (10%) and ovary (0%) cancers, suggesting a highest expression of TCTN2 in colorectal cancer (Table 1). TCTN2 association with colon cancer was also confirmed by using Receiving Operator Characteristic curves analysis (Supplementary Figure 2).

**Table 1:** IHC staining of colon, lung and ovary samples using the anti-TCTN2 mAb

IHC score	> 0			> 100		
*Colon cancer (%)*	30/47 (**64**)	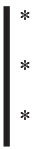	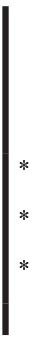	14/47 (**30**)	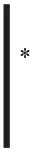	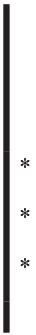
*Lung cancer (%)*	9/50 **(18)**			5/50 **(10)**		
*Ovary cancer (%)*	8/50 **(16)**			0/50 **(0)**		

Asterisk represents the statistical significance of Fisher's exact test. P value < 0.0001; ^***^; P value < 0.05. ^*^

To further validate these findings and to investigate the role that TCTN2 might have during cancer progression, the protein expression was further assessed on an independent collection of colon cancer cases (163 samples) selected on the basis of the availability of relevant clinical and molecular data (Supplementary Table 1). The TCTN2 protein was detected in 93% of the cases (21.5% with a strong or moderate staining), with no remarkable differences in detection frequency among tumor staging and grading histological criteria. (Table 2). The fraction of samples with moderate/strong TCTN2 staining moderately decreases in stage 4 and grade 3 cancers than in less advanced and low grade cancers (Table 2). All these data indicate that TCTN2 is associated with human cancers among which the highest expression level was found in colon cancer.

**Table 2:** TCTN2 frequency in CRC using an anti-TCTN2 mAb

Clinical Parameter	Status/Score	Total Positive (%)	Medium-Strong Positive (%)	
**Histology**	*adenocarcinoma*	134/141 **(95)**	34/141 **(24)**	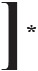
	*mucinous*	19/22 **(86)**	1/22 **(4)**	
**Stage**	*1*	27/27 **(100)**	8/27 **(30)**	
	*2*	43/47 **(91)**	10/47 **(21)**	
	*3*	52/53 **(98)**	13/53 **(24)**	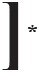
	*4*	27/32 **(84)**	4/32 **(12)**	
**pT**	*1*	10/10 **(100)**	1/10 **(10)**	
	*2*	30/31 **(97)**	8/31 **(26)**	
	*3*	90/95 **(95)**	24/95 **(25)**	
	*4*	24/28 **(86)**	2/28 **(7)**	
**pN**	*0*	74/79 **(94)**	19/79 **(24)**	
	*1*	45/47 **(96)**	11/47 **(23)**	
	*2*	29/32 **(91)**	5/32 **(16)**	
**Grading**	*1&2*	112/119 **(94)**	30/119 **(25)**	
	*3*	41/44 **(93)**	5/44 **(11)**	
**Metastasis**	*No*	94/99 **(95)**	25/99 **(25)**	
	*Yes*	62/67 **(92)**	10/67 **(15)**	
**Vascular Invasion**	*No*	41/44 **(93)**	9/44 **(20)**	
	*Yes*	111/118 **(94)**	26/118 **(22)**	
**Sex**	*female*	81/87 **(93)**	22/87 **(25)**	
	*male*	75/79 **(95)**	13/79 **(16)**	

Fisher's exact test P value < 0.05 is represented by asterisk ^*^

The specificity of the monoclonal and polyclonal antibodies used for IHC was verified by Western blot on HeLa cells transfected with full-length TCTN2. TCTN2 is predicted to encode a protein of 77 kDa with four potential glycosylation sites. As shown in Figure 1B, both polyclonal and monoclonal antibodies specifically detected a main band around 100 kDa in TCTN2-transfected HeLa cells, which was not visible in HeLa cells transfected with the “empty” plasmid. However, when the protein extracts were treated with PNGase F, the immune-reactive band shifted to around 80 kDa thus confirming the predicted molecular weight and glycosylation.

### TCTN2 protein is expressed in cancer cell lines and mainly localized on the intracellular side of the plasma membrane

**Figure 2:**
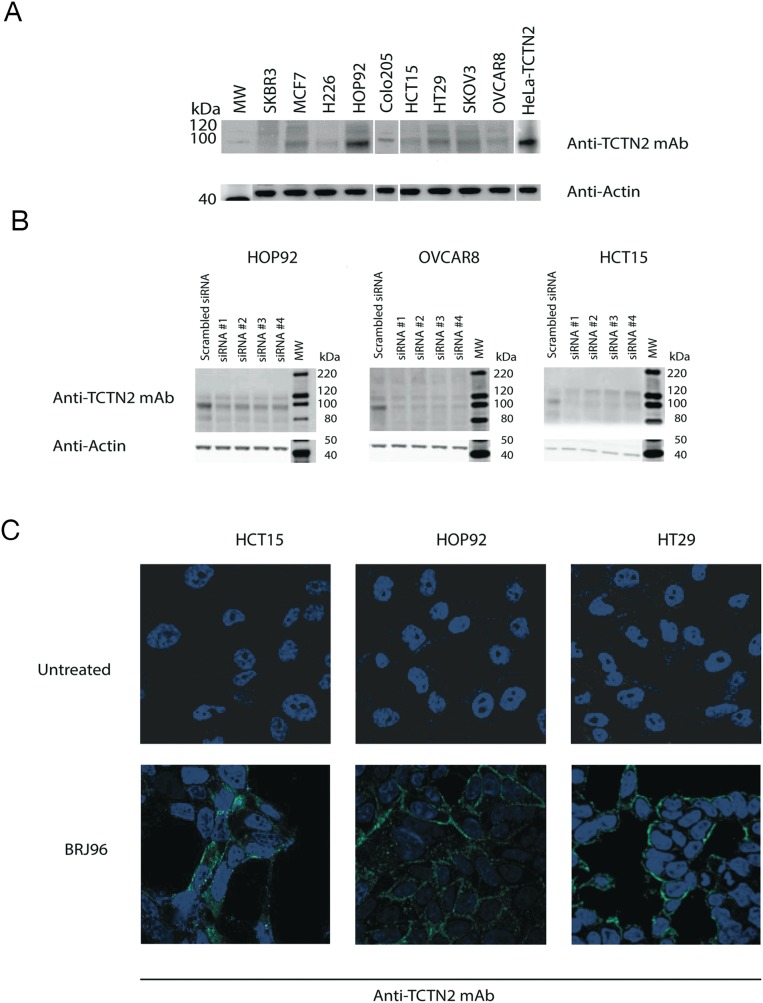
TCTN2 is expressed in cancer cell lines and localizes in the inner side of plasma membrane **(A)** Total proteins were extracted from breast (SKBR3 and MCF7), lung (H226 and HOP92), colon (HCT15, HT29 and Colo205) and ovarian (SKOV3 and OVCAR8) cancer cell lines. Proteins were resolved by SDS-polyacrylamide gel electrophoresis and analyzed by Western blot with anti-TCTN2 monoclonal antibody. Anti-β-Actin monoclonal antibody was used as a loading control. **(B)** TCTN2 was silenced in HOP92, OVCAR8 and HCT15 cancer cell lines with four commercially available (QIAGEN) TCTN2-specific siRNAs at 10 nM concentration or irrelevant siRNA (AllStars Negative Control siRNA, QIAGEN) using the Hi-Perfect transfection reagent (QIAGEN). TCTN2 expression was assayed 48 hours later by Western blot. Anti-β-Actin monoclonal antibody was used as a loading control. **(C)** TCTN2 localizes in the inner side of plasma membrane in cancer cells lines. HCT15, HOP92 and HT29 cells were analyzed by confocal microscopy. Cells were incubated with the anti-TCTN2 monoclonal antibody, with (lower panels) or without (upper panels) permeabilization pre-treatment with 0.01% BriJ96^®^. Cells were subsequently stained with Alexafluor 488-labeled goat anti-mouse antibodies to detect TCTN2 (green) and DAPI to visualize nuclei (blue).

To better characterize TCTN2 protein expression and localization in tumors we selected a panel of breast, lung, colon, and ovary tumor cell lines. In these cell lines we assessed TCTN2 expression by Western blot. Immuno-reactive bands around 100 kDa, were detected in MCF7, H226, HOP92, HCT15, HT29, Colo205, OVCAR8 and SCOV3 cell lines while the same bands were very faint or negative in SKBR3 (Figure 2A). These bands might likely represent different glycosylation states of the protein. To further evaluate the mAb specificity for the target we used four different TCTN2-specific siRNAs (10 nM) to silence TCTN2 expression in HOP92, OVCAR8 and HCT15 cells. All cell lines showed a significant reduction of TCTN2, both at transcript and protein levels, as verified by q-RT-PCR and Western blot with all tested siRNA, compared to cells treated with an irrelevant siRNA (Figure 2B). Afterwards, we assessed TCTN2 localization by confocal microscopy in HCT15, HOP92 and HT29 cancer cell lines, by permeabilizing or not the cell membrane with the detergent BRJ96. TCTN2 was detected at the level of the plasma membrane only in permeabilized cells whereas it was undetectable without detergent treatment (Figure 2C) Similar data were obtained in MCF7 and OVCAR8 cell lines (data not shown). This result indicates that the protein is not accessible to the antibody on the cell surface without permeabilization, thereby it is likely localized on the intracellular side of plasma membrane.

### Downregulation of TCTN2 by epigenetic editing

**Figure 3:**
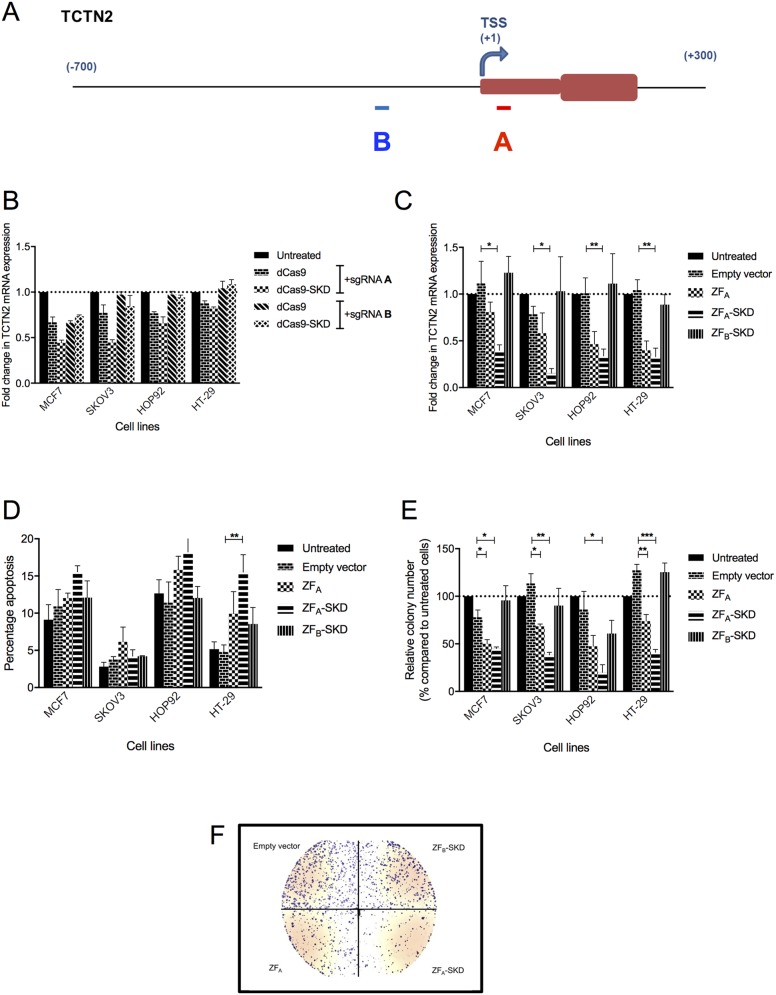
TCTN2 downregulation, by different platforms, alters growth phenotypes in four cell lines **(A)** Representation of TCTN2 gene promoter and first exon, transcription start site (TSS); A and B mark the region targeted. **(B)** TCTN2 mRNA downregulation using CRISPR-dCas9 sgRNA A and B regions alone. **(C)** TCTN2 mRNA downregulation using ZF A and B regions fused to a transcriptional repressor (SKD). **(D)** Apoptosis was measured using the 1, 1′,3,3,3′,3′-Hexamethylindodicarbocyanine iodide (DilC) assay in cells treated with different ZFs after TCTN2 downregulation. **(E)** Colony-forming assay in cells after TCTN2 downregulation. **(F)** Visual representation of the colony-forming assay from HT-29 cells. Data represent the mean value of two independent experiments, run in triplicate, and are shown as mean ± s.e.m. Statistical significance was assessed by non paired two-tailed Student's t-test (^*^P<0.05, ^**^P<0.01, ^***^P<0.001).

To investigate the alteration of cell growth and viability caused by loss of TCTN2 expression we developed epigenetic editing methods relying on the repression of its endogenous genomic locus and therefore able to perform a more sustained manner of silencing. To identify the best region to target within the promoter area, we used the CRISPR-dCas system to test two different sgRNAs recognizing regions: one mapping in the close proximity of the predicted transcription start site (TSS) and one 234 nucleotides upstream of the TSS (referred to as region A and B, respectively) (Figure 3A). In four cancer cell lines from different tissue origin (MCF7, breast cancer; SKOV3, ovary cancer; HOP92, lung cancer; HT29, colon cancer), we observed that the region A, the one located closer to the transcription start site, was better to achieve gene downregulation, irrespective of the effector domain used (Figure 3B). To further confirm our findings, we designed two DNA binding domain Zinc finger proteins to bind the same two regions of the promoter (referred as ZF_A_ and ZF_B_, respectively), fused to the transcriptional repressor Super Krab Domain (SKD). The same four cancer cell lines were transduced with retroviral vectors expressing ZF_A_, ZF_A_-SKD, or ZF_B_-SKD and 4 days later RNA expression level was assessed by q-RT-PCR and compared with cells treated with the empty vector, which served as negative control. ZF_A_-SKD transduced cells showed a significant reduction of transcription (ranging from 2 to 10-fold) in the four cell lines, whereas TCTN2 transcription levels in ZF_B_-SKD expressing and control cells was similar (Figure 3C). ZF_A_ alone also affected mRNA expression (from up to 2-fold) in HOP92 and HT29, although not significantly.

We then assessed whether enforced TCTN2 downregulation influences viability and growth of cancer cells. We first analyzed the apoptotic phenotype of the four cancer cell lines transduced with ZF_A_-SKD, ZF_B_-SKD, or ZF_A_ using the DilC1 staining assay [28], as compared to controls treated with the empty plasmid or untreated. Transduction with ZF_A_-SKD significantly increased apoptosis in HT-29 cells (approximately 3 fold) (Figure 3D). Interestingly in the same cells, also transduction with ZF_A_ alone increased the apoptotic phenotype, although it was not statistically significant. No significant effects on apoptosis were observed in the other cell lines and the expression of ZF_B_-SKD caused no effect in either cell line. Afterwards, we assessed cell proliferation using the PrestoBlue assay. A marginal reduction (approximately 20%) was observed in the proliferation of HT29 cells transduced with ZF_A_-SKD and ZF_A_ constructs while there was no significant effect on proliferation of the HT29 cells transduced with the ZF_B_-SKD construct when compared to the original cell line (data not shown).

### TCTN2 modulation influences cell invasiveness and anchorage-independent growth of cancer cells

**Figure 4:**
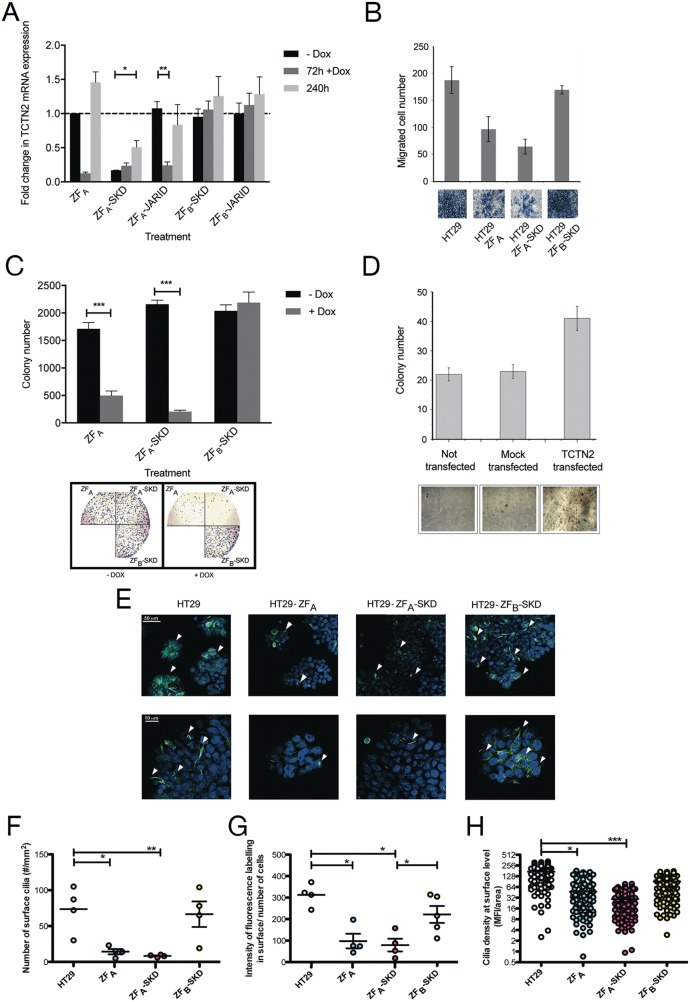
TCTN2 act as an oncogene: evidences collected by means of epigenetic editing and overexpression **(A)** Stable inducible HT-29 cells were generated and TCTN2 downregulation was addressed over time, after short-term induction of ZFP fusions. **(B)** Boyden invasiveness chamber assay in cells after TCTN2 downregulation induced by Dox addition. Cells migrated towards the lower surface of the chamber filters were fixed and counted after Diff-Quick staining. Images of the visual counting of each sample are reported below the graphs. **(C)** Colony forming assay in cells after TCTN2 downregulation induced by Dox. Lower panel: visual representation of the colony-forming assay from HT-29 cells without and with Dox treatment. **(D)** Anchorage-independent growth of HCT15 cells after transfection with a plasmid encoding full-length TCTN2. Data represent the mean value of two independent experiments, run in triplicate, and are shown as mean ± s.e.m Statistical significance was assessed by nonpaired two-tailed Student's t-test, (^*^P<0.05, ^**^P<0.01, ^***^P<0.001). **(E-H)** Confocal microscopy evaluation of cilia formation in Dox-treated HT-29 and ZFP fusions transduced cells. Cilia were labeled for alpha-tubulin acetylation (green) and nuclei were stained with DAPI (blue). Representative images at lower (E, top row; scale bar: 50 μm) and higher (E, bottom row; scale bar: 10 μm) magnification are shown for HT-29 cells and ZFP fusions transduced cells, as specified on top of the panels. White arrow-heads point to labeled cilia. Image analysis was conducted on n=2 independent biological replicates, accounting for n=650 cells over at least 8 FOVs/condition. Results in HT-29 cells and ZFP fusions transduced cells, were respectively plotted as number of surface cilia/area (F panel), surface fluorescence intensity/number of cells (G panel), and cilia density (MFI/area) (H panel). Statistical significance was attested using ANOVA test plus Dunnet comparisons (^*^P<0.05, ^**^P<0.01, ^***^P<0.001).

We then investigated the anchorage-independent growth phenotype of the transduced cell lines by the soft agar assay, one of the most stringent test commonly used to assess malignant transformation in cells. All cell lines expressing ZF_A_-SKD showed a reduced number of colonies in the soft agar assay (ranging from 2 to 5 fold). Also in this assay, cells treated to express ZF_A_ alone showed a reduced capacity to produce colonies (Figure 3E–3F). The observed phenotypic effects were further confirmed by creating stable cell lines able to downregulate TCTN2 from the endogenous genomic locus with an inducible system. To this aim, the HT-29 cell line was transduced with a doxycycline (Dox) inducible system with different DNA binding domains coupled to either a transcriptional repressor (SKD) or an epigenetic enzyme (JARID1A) that demethylates H3K4me3. After adding 100 μg/ml of Dox for 72 hours to the cells, an efficient downregulation of TCTN2 in stable HT-29 cells was achieved using ZF_A_, ZF_A_-SKD and ZF_A_-JARID, whereas it was unaltered by ZF_B_-SKD and ZF_B_-JARID constructs (Figure 4A). TCTN2 was also in part downregulated before Dox addition, likely due to a leaky expression of ZF_A_-SKD. Despite potential leakiness, sub-culturing cells for 7 additional days without Dox reversed the TCTN2 downregulation caused by either ZF_A_ constructs.

The influence of TCTN2 downregulation on tumor aggressiveness was assessed in HT-29 cells using *in vitro* assays measuring features like cell invasiveness and clonal growth in anchorage independent conditions. In the Boyden assay, also useful to measure cell migration, the cells transduced with the ZF_A_-SKD construct showed a marked reduction of invasiveness (approximately 3 fold) after Dox treatment (Figure 4B). Cells modified with ZF_A_ also showed reduced invasive properties, as compared to cells transduced with the ZF_B_-SKD or untreated HT-29 cells.

The soft agar assay was used to assess clonal growth. After Dox treatment, HT29 cells transduced with both the ZF_A_ constructs showed a marked decrease in the clonogenic phenotype (up to 1 log reduction in the number of colonies, compared to cells cultured without Dox) while cells transduced with the ZF_B_-SKD construct were not affected by the Dox treatment (Figure 4C).

We then sought to confirm the oncogenic potential of TCTN2 by over-expressing it in human cells and determining clonal growth. HCT15 cells were transiently transfected with a TCTN2-encoding plasmid and the resulting phenotype was monitored for 7 days in the soft agar assay, by assessing the number of cell colonies, as compared to cells transfected with the empty plasmid. As shown in Figure 4D, TCTN2 over-expression resulted in an increase of the number of HCT15 cells able to form colonies (approximately 2 fold), as compared to control cells transfected with the empty vector.

### TCTN2 downregulation impairs the formation of primary cilia

Given the peculiar cellular localization assigned to TCTN2, it was very intriguing to assess if TCTN2 loss of expression could have also an influence in cilium formation. To this aim we monitored the presence of acetylated α-tubulin, which is essential for the assembly of primary cilia. After 72 h of Dox treatment, HT-29 cells and the relative ZF-transduced cell lines were permeabilized, stained with an anti-acetylated α-tubulin antibody and analyzed by confocal microscopy. As shown in Figure 4E-4H a marked impairment of cilia formation was observed in the ZF_A_-SKD transduced cells compared to untreated HT-29 cells in terms of both numbers of surface cilia (14±3/mm2 vs. 73±15/mm2, P<0.01) and density of acetylated α-tubulin associated to the cilium quantified as cilia mean fluorescence intensity relative to the occupied area (47±4 vs. 171±6, P<0.001). Similar effects were observed when comparing ZF_A_-SKD to ZF_B_-SKD modified cells (P<0.05 for surface cilia number/area; P<0.001 for cilia density). Again cells treated to express the ZF_A_ construct also showed a visible reduction of both surface cilia and cilia density, compared to untreated cells (P<0.05) and to ZF_B_-SKD modified cells (P<0.05 and P<0.001, respectively).

## DISCUSSION

TCTN1, TCTN2 and TCTN3 (TCTNs) proteins are emerging as critical ciliary components that form high affinity complexes in the cilium transitional zone. They could modulate signal transduction of Hh and Wnt pathways through their functional interaction with MKS1 [20]. While the relevance of Wnt and Hh in cancer is well documented, the role of TCTNs has been so far marginally addressed. The only member of this family that has been reported to act as an important player in human cancers is TCTN1 [29]. Indeed, it is expressed in human glioma, pancreas and prostate cancer cell lines. Moreover, in human glioblastoma (GBM) TCTN1 overexpression predicts poor clinical outcome for patients [30].

The present study significantly contributes to the current knowledge on TCTN2, by providing robust experimental evidence that it is associated with human cancer. By an IHC study we discovered that the protein is highly expressed in colon, lung, and ovary cancers, among which the highest expression level (based on samples with IHC score >100) was found in colon cancer. The presence of TCTN2 in human cancer is also supported by transcription profile data available in the Oncomine database (https://www.oncomine.org) reporting that this gene is upregulated in specific cancer subsets among colorectal cancer, non small cell lung cancer, gastric cancer, sarcoma, lymphoma and others.

We found that TCTN2 is expressed in cell lines derived from different cancers, and it is associated to the plasma membrane, but not protruding towards the extracellular side. Notwithstanding our IHC data clearly showed a main TCTN2 association with colon cancer, we did not found any remarkable peak of TCTN2 expression in colon cell lines vs the other tested cancer cell lines. It would be interesting to investigate whether TCTN2 expression in colon cancer could increase in response to specific signal or environmental conditions.

In this study we demonstrated that TCTN2 plays an important role in the growth of cancer cells by using targeted epigenetic editing. Indeed, loss of TCTN2 reduced the ability of four cancer cell lines to form colonies independently of a solid surface and, remarkably, it increased cell apoptosis; it also influences cell viability, though to a moderate extent. Moreover, transient TCTN2 over-expression in HCT15 cells elicited by conventional cDNA transfection substantially increased the colony-forming phenotype. Additionally, we found that loss of TCTN2 caused by transient silencing significantly affects cell invasiveness, suggesting a role for the protein in the development of the tumor cell ability to penetrate the surrounding tissues leading to the formation of metastasis. Further *in vivo* investigation are needed to confirm the tumorigenicity and metastasis formation capacity of tumor cell lines with intact or downmodulated TCNT2 expression. Interestingly, we found that TCTN2 is expressed in the plasma membrane of cancer cell lines probably associated to the cilium. Indeed we observed that loss of TCTN2 expression significantly reduces cilia formation in HT-29 cells. An interesting aspect to be elucidated is whether the cilium defect caused by loss of TCTN2 expression also contributes to the altered migratory/invasive phenotype of colon cancer cells.

Moreover, we hypothesize that the phenotypic effects caused by variation of TCTN2 expression could be due to a chain signaling reaction mediated by MSK1. As described for TCTN1, variation of TCTN2 expression could affect the interaction of the TCTN complex with MSK1 and influences its expression in the cilia, thereby leading to Wnt and Shh signaling transduction that can ultimately result in aberrant growth of cancer cells. This aspect deserves future studies.

Overall, our study provides additional hints on the role of TCTNs proteins in cancer development. Knock down approaches proved that TCTN1 is essential for cancer cell viability and proliferation, suggesting that its dysregulation may play a key role in tumorigenesis [23, 31, 32]. TCTN2, taking part to a supramolecular functional complex with TCTN1, could act in combination with it in conferring cellular phenotypes relevant for tumor growth and spread to distal sites. Indeed, the capability to grow in an anchorage independent way is considered a hallmark of carcinogenesis our experimental evidences showing that loss of TCTN2 expression significantly reduces such phenotypes suggest that it might act as an oncogene. Moreover, it would be interesting to test whether, as reported for TCTN1 in GMB [30], TCTN2 could represent a prognostic factor for TCTN2-over-expressing cancers. Finally, our *in vitro* data also provide the rationale for the design of novel TCTN2-targeting drugs. Targeted epigenetic editing can eventually serve as a therapeutic approach to stably alter gene expression patterns in cells [33, 34].

## MATERIALS AND METHODS

### Reagents and cell cultures

Unless specified, all reagents were obtained from Sigma. His-tagged recombinant TCTN2 domains and polyclonal antibodies were generated in E. coli as described [26]. Human cells were obtained from the ATCC collection and, unless differently stated, cultured under ATCC recommended conditions. Cell culture media were from BioWhittaker (Walkerville, MD, USA). All cells were cultured at 37°C under 5% *CO_2_.*

### IHC analysis

TMA production and IHC staining were performed essentially as previously described [26, 35, 36]. Briefly, formalin-fixed, paraffin-embedded tissue blocks of CRC resections and normal samples were retrieved from the archives of the Institute of Pathology, University Hospital Basel and the Institute of Clinical Pathology Basel, Switzerland. CRC and normal samples were arrayed in parallel on the same TMA slides and analyzed simultaneously. One tissue cylinder with a diameter of 0.6 mm was punched from morphologically representative tissue areas, mostly central tumor areas and rather away from the infiltrating tumor border. Clinico-pathologic data from the corresponding series of cancer cases were obtained from archived files. Concerning the scoring of TCTN2 staining, slides were screened semi-quantitatively for the percentage and the intensity of the signal for TCTN2. At least 100 cells were counted for each punch. Intensity of the signal was graded semiquantitatively in 4 groups from 0 (no positivity) to 3 (strong positivity). A case was considered low positive if showed a positive signal between 10% and 33% of cells, moderate positive between 33 and 66% and strong positive more than 66%. Negative control samples were prepared by using an irrelevant isotype control antibody and/or by omitting the primary antibody. Ethics approval for this study was obtained from the Ethic Committee for Human Research of the Basel University Hospital (Ethikkommission Nord-und Zentralschweiz, EK 322/13).

### Cell transfection

A pcDNA3.1D (Invitrogen) derivative plasmid encoding TCTN2 full-length cDNA was generated and sequence verified. HeLa or HCT15 cells (400,000/well, in 6-well plates) were transfected with 4 micrograms of the TCTN2 plasmid or with the empty vector as negative control using the Lipofectamine-2000 transfection reagent (Invitrogen) following the manufacturer's protocol.

### TCTN2 silencing with siRNA technology

TCTN2 was silenced in HOP92 and OVCAR8 cancer cell lines with four commercially available (QIAGEN) TCTN2-specific siRNAs at 10 nM concentration (#1- SI04153632: TGCATCCGTCCAGTTTATTAA), (#2- SI04159183: AAGCCTATAGTTAGACAACCA), (#3- SI04200686: TTGGAACTATACCAAGAACGA), (#4- SI04220433: TGGCTCGAAATAATACGTGTA), or irrelevant siRNA (AllStars Negative Control siRNA, QIAGEN) using the Hi-Perfect transfection reagent (QIAGEN) following the manufacturer's protocol. TCTN2 expression was assayed 48 hours later by real-time q-PCR and Western blot with anti-TCTN2 monoclonal antibody. Anti-β-Actin monoclonal antibody was used as a loading control.

### RNA extraction and q-RT-PCR analysis

RNA extraction from cell lines was performed using the RNeasy mini kit (QIAGEN) according to manufacturer's protocol and 500 ng of it were reverse transcribed using Superscript III Reverse Transcriptase (Life Technologies) with oligo-dT. Triplicate cDNA samples from each sample (equal to 50 ng RNA/sample) were subjected to to assess the expression of TCTN2 (primers RT^2^ qPCR Primer Assay for Human TCTN2, QIAGEN) using the Quantitect SYBR Green PCR kit (QIAGEN). MAPK or GAPDH (primers RT^2^ qPCR Primer Assay for Human MAPK or GAPDH, QIAGEN) were used as an internal normalization controls, respectively. Data were analyzed with the One-Step Plus q-RT-PCR equipment (Applied Biosystems). Fold change in mRNA expression above control untreated cells was calculated based on the cycle threshold (ΔΔCt) method after normalization to MAPK or GAPDH expression.

### Electrophoresis and western blot analysis

Cell monolayers were detached with PBS-0.5 mM EDTA and lysed with either Triton X-100 or RIPA buffer by incubating cell suspensions for 30 min at 4 °C with continual rotation prior to removal of the insoluble fraction by centrifugation at 10000xg for 30 min at 4 °C. Total protein extracts were loaded on SDS-PAGE (2×105 cells/lane) and expression of target proteins was assessed by Western blot analysis using specific antibodies. Western blot was performed by separation of the protein extracts on pre-cast SDS-PAGE gradient gels (NuPage 4-12% Bis-Tris gel, Invitrogen) under reducing conditions, followed by electro-transfer to nitrocellulose membranes (Invitrogen) according to the manufacturer's recommendations. The membranes were blocked in blocking buffer composed of 1x PBS-0.1% Tween 20 (PBST) added with 10% dry milk, for 1 h at room temperature, incubated with the antibody diluted 1:2500 in blocking buffer containing 1% dry milk and washed in PBST-1%. The secondary HRP-conjugated antibody (goat anti-mouse immunoglobulin/HRP, Perkin Elmer) was diluted 1:5000 in blocking buffer, and chemiluminescence detection was carried out using a Chemidoc-IT UVP CCD camera (UVP) and the Western LightningTM Cheminulescence Reagent Plus (Perkin Elmer), according to the manufacturer's protocol.

### Confocal microscopy analysis of TCTN2 expression

Cells were plated on glass coverslips. After 48 h, they were washed with PBS and fixed with 3% formaldehyde solution in PBS for 20min at RT. Then, after extensive washing in PBS, the cells were permeabilized with 0.01% BriJ96® (Fluka) and subsequently incubated with the anti-TCTN2 mAb antibody anti-overnight at 4 °C. Cells were then stained with Alexafluor 488-labeled goat anti-mouse antibodies (Molecular Probes). DAPI (Molecular Probes) was used to visualize nuclei. The cells were mounted with glycerol plastine and observed under a laser-scanning confocal microscope (LeicaSP5) and Z stacks of fields of cells were acquired from each condition using a 63x oil objective (N.A. 1.4). Digital images were taken using LAS AF software (Leica).

### Engineering ZFPs and plasmid construction

Plasmids containing a mammalian codon-optimized dCas9-VP64 activator (pMLM3705) and the single-chain guide RNA encoding plasmid (pMLM3636) were bought from Addgene. An additional multiple cloning site was added by replacing the VP64 activator in the dCas9-VP64 with a sequence containing a PacI restriction site (new plasmid referred to as dCas9-Empty). The Super Krab Domain (SKD) was subcloned from pMX-6ZF-SKD into dCas9-VP64, by using BamHI and XhoI enzymes, to replace VP64 with SKD. Two target regions of 20 bps of the TCTN2 promoter were selected to design gRNAs based on close proximity to the transcription start site (TSS) (A: ; B:). Cloning of gRNAs was achieved as previously described. Briefly, pairs of DNA oligonucleotides encoding 20 nucleotide gRNA targeting sequences were annealed together to create double-stranded DNA fragments with 4-bp overhangs. These fragments were ligated into BsmBI-digested plasmid pMLM3636. Two target regions of 18 bps of the TCTN2 promoter close to the gRNA binding sites were selected based on high affinity predictions (www.zincfingertools.org) and the uniqueness of the target sites confirmed by a blast on NCBI. Double stranded DNA oligos (BIO BASIC, Markham, Canada) coding for the two 6-finger ZFPs (ZF_A_: GCAACAGGGGGCGGGGGG and ZF_B_: GGGGAGGAAGCCGCAGCC minus strand) flanked with the restriction site SfiI, were subcloned into the pMX-IRES-GFP alone called no effector domain (NED) or containing either the gene repressor super krab domain (SKD) or epigenetic effector domain JARID1a.

### Retroviral transductions

HEK293T cells were co-transfected with the retroviral vector pMX-IRES-GFP along with VSV-G viral envelope (pMD2.G) and the gag/pol proteins (pMDLg/pRRE) using CaPO_4_. 48 and 72 hours after transfection, the viral supernatant was used to transduce host cells supplemented with FBS and 5μg/ml polybrene (Sigma, St. Louis, MO, USA). Cells were harvested for further experiments three days after the last transduction. GFP positivity of cells was assessed on a Calibur Flow Cytometer (Beckton Dickinson Biosciences).

### Generation of stable cell lines

Retroviral particles from pRetroX-Tet-On-Advanced (pTet-On) (CloneTech, Mountain View, CA) were generated using conventional CaPO_4_ transfection of HEK-293T. Virus-containing supernatant was harvested 48 and 72 hours post-transfection, supplemented with FBS and 5μg/ml polybrene, and used to transduce HT-29 cells. Two days after transduction, cells were selected with 1μg/ml geneticin (Gibco/Invitrogen) for 5 days and individual clones were subcultured for testing using the pRetroX-Tight-Luc-Pur (CloneTech, Mountain View, CA). The clone with the highest expression of luciferase after induction was chosen for subsequent use. The coding region of the fusion proteins of ZF_A_ and ZF_B_ with SKD or JARID1a were subcloned into the expression vector pRetroX-Tight-Pur (CloneTech, Mountain View, CA) using the BamHI/NotI restriction sites. Retroviral transduction of the plasmids was carried out as described previously using the stable pTet-On HT-29 cells. Two days after transduction cells were selected with 1 μg/ml geneticin (Gibco/Invitrogen) for 10 days. Expression of the fusion proteins was induced using Doxycycline (Dox, 100 μg/ml) for 72 hours.

### Clonogenic assay

Following transfection or transduction, cells were plated in 6-wells plates (2000-4000 cells per well). After two weeks, medium was aspirated and colonies were stained with Coomassie brilliant blue (Bio-Rad). The number of colonies was determined using phase contrast microscopy and Image J.

### Apoptosis assay

Cell apoptosis was measured using the 1, 1′,3,3,3′,3′-Hexamethylindodicarbocyanine iodide (DilC) assay (Enzo Life Sciences) and the Annexin V-PI assays (Sigma) according to the manufacture's protocol. For the DilC assay, following treatment, cells were trypsinized and incubated in culture medium supplemented with DilC (50 nM) for 15 min at 37°C. After washing with PBS, DilC signal was analyzed using FACS Calibur (BD Biosciences). The percentage of apoptotic cells was determined as the number of viable cells with decreased DilC intensity, as reported before [37]. Assays were performed in 96w-well plates according to the manufacturer's instruction. Each experiments was carried out in triplicate and averaged from at least 3 independent experiments.

### Cell invasiveness

Cell invasiveness was assessed by the Boyden chamber assay. Cells (2000/well) were placed onto Matrigel-coated 24-well plates and, after ON incubation at 37°C, non-invading cells were removed mechanically using cotton swabs, and micro-porous membrane containing the invaded cells was fixed in 96% methanol and stained with Diff-Quick staining solutions. Invasiveness was evaluated by counting the cells that migrated towards the lower surface of the filters (10 randomly chosen fields for each filter). Each experiment was carried out in triplicate and averaged from at least 3 independent experiments.

### Cell proliferation

PrestoBlue cell viability reagent (Life Technologies) was used to measure cell proliferation. Briefly 6-, 20- and 60 x10^3^ cells were seeded per well in triplicate in 96-well white plates in 90μl medium. Cells were incubated for 72 h. Then 10μl of PrestoBlue was added to each well. Cells were further incubated for 1 h. Cell proliferation was assessed by detecting fluorescence at 590 nm.

### Cilia formation

The formation of cellular cilia was assessed via confocal laser scanning microscopy imaging of cellular samples labeled for alpha-tubulin-acetylation using the mouse anti-acetylated alpha-tubulin (Abcam Ab24610) as primary mAb followed by secondary labeling with Alexa-488 conjugated anti-mouse antibody, and counter-stained with DAPI for nuclear evaluation and cell count. Independent biological replicates of 72-h Dox treated control HT-29 and ZF-transduced cell lines were observed acquiring at least 5 field-of-view at lower magnification (40x, NA 1.40) and 3 field-of-view at higher magnification (63x, NA 1.40, zoomed 2x) using a SP5 true confocal laser scanning microscopy (Leica microsystems), accounting for n=650 analyzed cells. Z-volumes of around 15 μm were acquired to have a complete 3D rendering of cellular samples. 16-bit images at 2048×2048 pixel rate were acquired for each Z-frame. For surface level cilia formation, analysis was specifically performed at surface Z-planes, evaluating the number of surface cilia per area and the density of cilia labeling (MFI/area). In parallel, the biological replicates were also acquired using a high-resolution wide field microscope (Nikon Instruments), equipped with cSMOS camera (ANDOR) for larger field of view analysis at surface level (8400×7500 pixel, 16-bit). Analysis were performed using NIS-elements 4.51 (Nikon Instruments) and FIJI [38].

### Statistical analysis

Statistical analysis of the phenotypic data was performed using two-tailed Student's t-test. The statistical association between the clinico-pathological variables and TCTN2 IHC staining was analyzed using the two-tailed χ^2^ test and Fisher's exact tests. For microscopy analysis, normally distributed data were assessed with ANOVA test followed by Dunnet comparisons. P- values ≤ 0.05 were considered significant. Statistical analysis was carried out with the use of GraphPad software. The AUC (Area Under the Curve), SE, SP and optimal cutoff were obtained by the CombiROC prediction tool [39].

## SUPPLEMENTARY MATERIALS FIGURES AND TABLE


