# Supplementary material for: TCTN2: a novel tumor marker with oncogenic properties
Source: Oncotarget. 2017 Aug 24;8(56):95256–69. doi: 10.18632/oncotarget.20438 (PMC5707019; doi:10.18632/oncotarget.20438)
Supplement: Supplementary file 1 [file oncotarget-08-95256-s001.pdf]

TCTN2: a novel tumor marker with oncogenic properties

SUPPLEMENTARY MATERIALS

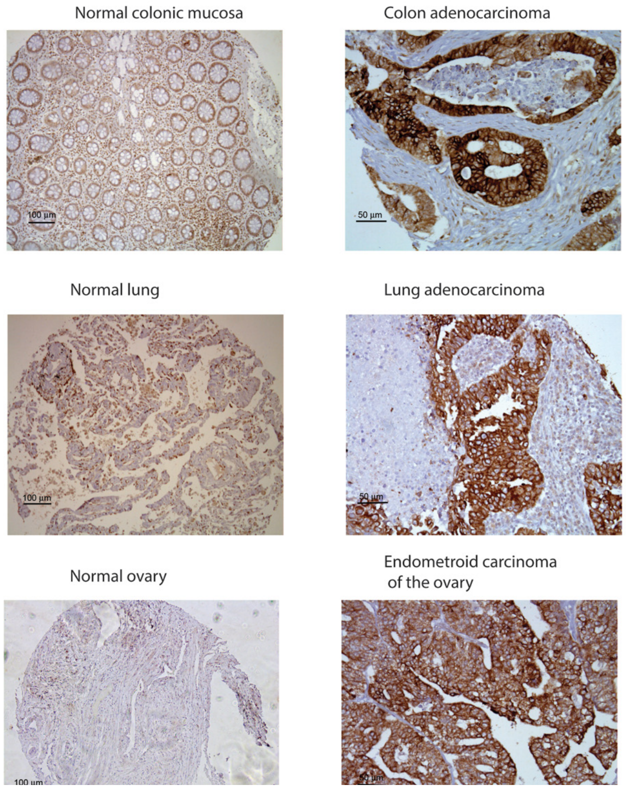

Supplementary Figure 1: TCTN2 is overexpressed in primary tumours of colon lung and ovary. TCTN2 is overexpressed in tumour samples and localises at the cell membrane. Tissue micro array (TMA) and IHC staining were performed as previously described [35]. Normal and cancerous samples from colon, lung and ovary were stained with the anti-TCTN2 polyclonal antibody YOM-291, arrayed in parallel on the same TMA slides and analysed simultaneously.

A

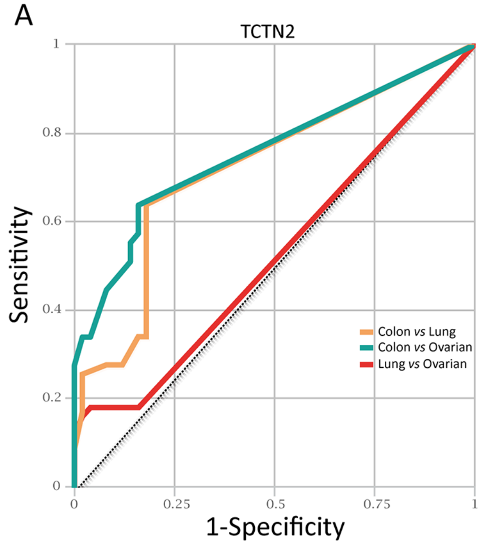

B

CTN2	AUC	SE	SP	Opt Cutoff
Colon *vs* Lung	0.720	0.638	0.820	0.415
Colon *vs* Ovarian	0.757	0.638	0.840	0.419
Lung *vs* Ovarian	0.523	0.180	0.960	0.592

C

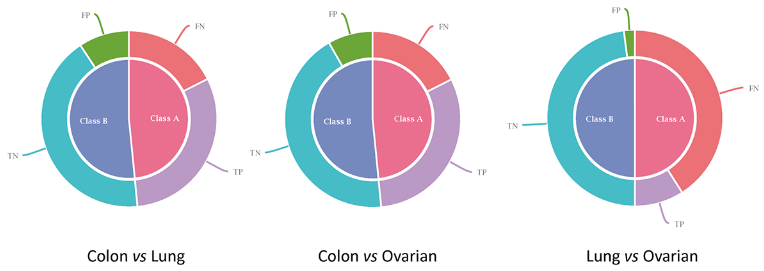

Supplementary Figure 2: ROC curve comparative analysis of TCTN2 detection in colon, lung and ovary cancer. (A) The ability of the anti-TCTN2 mAb to discriminate colon, lung and ovary cancer based on TCTN2 detection was analyzed by ROC curve plot. **(B)** Table summarizing relevant parameters of all compared curves. **(C)** Pie charts showing the fraction of predictions (FN: false negative; FP; false positives; TN; true negative; TP; true positive).

Supplementary Table 1: Description of the CRC TMA

	Clinical Parameter	Total samples
Histology	adenocarcinoma	141
	mucinous	22
Topography	ascendens	25
	cecum	27
	descendens	7
	rectosigmoid	12
	rectum	40
	sigmoid	36
	transversum	17
Stage	1	27
	2	47
	3	53
	4	32
pT	1	10
	2	31
	3	95
	4	28
pN	0	79
	1	47
	2	32
Grading	1&2	119
	3	44
Metastasis	No	99
	Yes	67
Vascular Invasion	No	44
	Yes	118
Sex	female	87
	male	79
